# Correcting common misconceptions to inspire conservation action in urban environments

**DOI:** 10.1111/cobi.13193

**Published:** 2018-10-24

**Authors:** Kylie Soanes, Michael Sievers, Yung En Chee, Nicholas S. G. Williams, Manisha Bhardwaj, Adrian J. Marshall, Kirsten M. Parris

**Affiliations:** ^1^ School of Ecosystem and Forest Sciences The University of Melbourne VIC 3010 Australia; ^2^ School of BioSciences The University of Melbourne VIC 3010 Australia

**Keywords:** cities, conservation policy, novel habitats, patch size, urban biodiversity, urban conservation, urban green space, área verde urbana, biodiversidad urbana, ciudades, conservación urbana, hábitats novedosos, políticas de conservación, tamaño de fragmento, 保护政策, 新生境, 斑块大小, 城市保护, 城市多样性, 城市绿地

## Abstract

Despite repeated calls to action, proposals for urban conservation are often met with surprise or scepticism. There remains a pervasive narrative in policy, practice, and the public psyche that urban environments, although useful for engaging people with nature or providing ecosystem services, are of little conservation value. We argue that the tendency to overlook the conservation value of urban environments stems from misconceptions about the ability of native species to persist within cities and towns and that this, in turn, hinders effective conservation action. However, recent scientific evidence shows that these assumptions do not always hold. Although it is generally true that increasing the size, quality, and connectivity of habitat patches will improve the probability that a species can persist, the inverse is not that small, degraded, or fragmented habitats found in urban environments are worthless. In light of these findings we propose updated messages that guide and inspire researchers, practitioners, and decision makers to undertake conservation action in urban environments: consider small spaces, recognize unconventional habitats, test creative solutions, and use science to minimize the impacts of future urban development.

## Abstract 2

*Article impact statement*: Conserving native biodiversity is both important and achievable in urban environments.

## Abstract (es)

Corrección de Ideas Erróneas para Inspirar Acciones de Conservación en Ambientes Urbanos

**Resumen**

A pesar de las repetidas llamadas a actuar, las propuestas para la conservación urbana con frecuencia se enfrentan a reacciones de sorpresa o escepticismo. Todavía existe una narrativa penetrante en la política, la práctica y el psique del público que dicta que los ambientes urbanos, aunque sean útiles para comprometer a las personas con la naturaleza o para proporcionar servicios ambientales, tienen poco valor para la conservación. Argumentamos que la tendencia de pasar por alto el valor para la conservación de los ambientes urbanos surge de las ideas erróneas sobre la habilidad que tienen las especies nativas para persistir dentro de ciudades y pueblos y que esto, en cambio, impide la acción efectiva de la conservación. A pesar de esto, la evidencia científica reciente muestra que estas suposiciones no siempre se sostienen. Aunque casi siempre es verdad que incrementar el tamaño, la calidad y la conectividad de los fragmentos de hábitat mejorará la probabilidad de que una especie pueda persistir, lo contrario, que los hábitats fragmentados, degradados y pequeños que se encuentran en los ambientes urbanos son inútiles, no lo es. A la luz de estos hallazgos proponemos mensajes actualizados que guíen e inspiren a los investigadores, practicantes y a los tomadores de decisiones a emprender acciones de conservación en ambientes urbanos: considerar espacios pequeños, reconocer hábitats poco convencionales, probar con soluciones creativas, y utilizar la ciencia para minimizar los impactos de desarrollos urbanos futuros.

## 摘要

尽管人们一再呼吁要采取城市保护行动, 然而却常常对相关提案表示诧异或怀疑。在政策制定、实践行动和公众心理中仍存在一种普遍的认识, 即城市环境虽然有助于人们亲近自然并提供生态系统服务, 但其保护价值微乎其微。我们认为, 这种忽视城市环境保护价值的倾向来源于对本地物种在城市和乡镇间续存能力的误解, 而这反过来又阻碍了有效的保护行动。然而, 最近的科学证据表明这些种种假设并不总是成立。虽然通常情况下增加生境斑块的大小、提高其质量和连接度可以提高物种续存的可能性, 但反过来看, 并不是说退化或碎片化的小面积生境在城市环境中就毫无价值。鉴于以上发现, 我们建议更新这些指导和激励研究者、实践者和决策者采取城市环境保护行动的信息, 应考虑小尺度空间的保护、认识非传统生境、尝试创新性解决方案, 并利用科学知识来减少未来城市发展带来的影响。 **【翻译: 胡怡思; 审校: 聂永刚】**

## Introduction

The value of urban environments for the conservation of native species can be surprisingly contentious. Recent global analyses indicate urban areas are expanding and are a major cause of biodiversity loss (Seto et al. 2012; Aronson et al. 2014). Urban areas encompass a wide range of ecosystems, include regions of high native biodiversity, and are inhabited by rare and threatened species (Schwartz et al. 2002; Rebelo et al. 2011; Kantsa et al. 2013; Ives et al. 2016). Given that urban areas are expanding and these areas contain important native species, it follows that protecting and promoting biodiversity in such areas should be critical. Yet in practice, progress is slow and uneven. Despite repeated calls to action in the scientific literature (e.g., Miller & Hobbs 2002; Rosenzweig 2003; Dunn et al. 2006), suggestions for urban biodiversity conservation are still met with surprise, doubt, or scepticism (Semlitsch & Bodie 1998; Sanderson & Huron 2011; Salomon Cavin 2013). It seems there remains a pervasive narrative in policy, practice, and the public psyche that urban environments, while useful for engaging people with nature or providing ecosystem services, are of little conservation value. We argue that this tendency to undervalue urban environments stems from misconceptions about the ability of native species to persist within cities and towns. Common assumptions are that the urban environment is not suitable for conservation in the long term due to the quantity and of remnant habitat, an inevitable extinction debt, and unmanageable impacts from human activity. These threats are real and the concerns legitimate, but they do not preclude meaningful conservation. We examined how these misconceptions are increasingly at odds with the findings of recent urban biodiversity research and propose updated narratives to inspire and guide conservation action.

## Misconceptions Underpinning Negative Views of Urban Conservation

Most conservation strategies and policies place a premium on large, high‐quality, well‐connected patches of remnant vegetation with a low prevalence of threats. However, such patches are rare in the urban realm and this invites the view that urban environments are inherently worse for conservation. The small, heavily modified habitats so common to urban environments are rarely protected by policy, vulnerable to death by 1000 cuts (Tulloch et al. 2016), and often considered expendable (Semlitsch & Bodie 1998). In other cases, urban areas are overlooked altogether. For example, it is not unusual for large‐scale conservation prioritization or planning exercises to exclude urban areas from consideration or assign them a low conservation value a priori (Moilanen et al. 2005). Policy makers, land managers, and conservation practitioners are, thus, reluctant to invest limited conservation dollars and effort in an area deemed to be of high risk, low value, and with a low probability of success (Miller & Hobbs 2002; Sanderson & Huron 2011; Olive 2014). But are these assumptions about urban environments supported by current research?

Although it is generally true that increasing the size, quality, and connectivity of habitat patches will improve the probability that a species can persist, the inverse is not that small, degraded, or fragmented habitats are worthless. Prugh et al.’s (2008) meta‐analytic study of >1,000 bird, mammal, reptile, amphibian, and invertebrate population networks on 6 continents demonstrated that patch area and isolation are surprisingly poor predictors of occupancy for most species. Further, a recent review found little evidence to support the notion that habitat fragmentation per se has a negative impact on biodiversity (Fahrig 2017). The role of the intervening matrix in providing resources and facilitating movement is now well recognized, to the point that it can no longer simply be referred to as nonhabitat (Franklin & Lindenmayer 2009; Driscoll et al. 2013).

Turning to organisms themselves, we note that the life‐history traits of a species, such as reproductive requirements, generation time, and mobility, can play a large role in determining the likelihood of persistence in urban environments. For example, large patches of habitat may not be required to support the persistence of small plants with limited dispersal ability (McCarthy et al. 2006). Other factors such as adaptedness and adaptive potential (i.e., phenotypic or behavioral plasticity) also influence the capacity of organisms to exploit and survive in urban environments (McDonnell & Hahs 2015). These research insights do not support the lost cause narrative so frequently applied to urban environments. The mismatch between common understanding (among researchers and practitioners) and recent scientific evidence (e.g., Norton et al. 2016) suggests the need for revised messages to guide conservation action in cities. We devised 4 key messages to correct common misconceptions that limit urban conservation action, identified examples from the growing body of research on urban biodiversity, and considered how policy and practice could be updated to make these actions more effective. In short, urban conservation must consider small spaces, recognize unconventional habitats, test creative solutions, and use science to minimize the impacts of future urban development.

## Messages to Inspire Urban Conservation

### Valuing Small Urban Spaces

**Figure 1:**
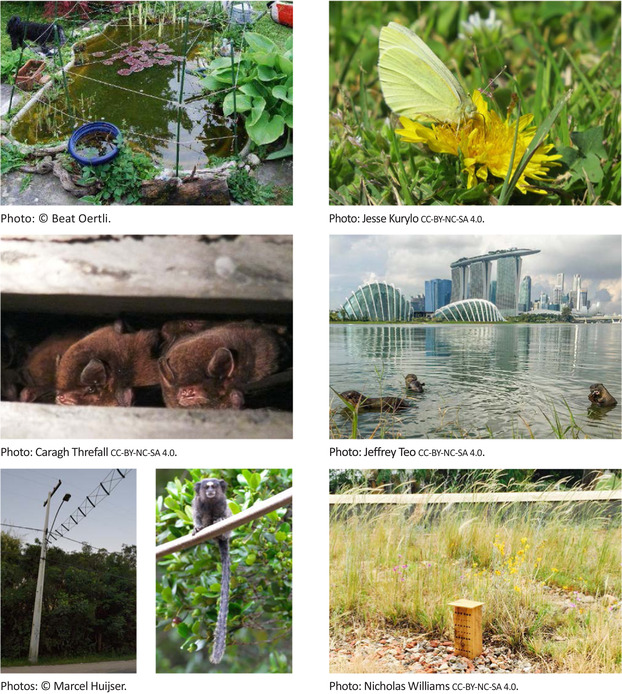
Important components of urban conservation from top left to bottom right: (a) small areas that provide habitat, pond in Geneva, Switzerland; (b) a benevolent matrix, road verge with common dandelion and a butterfly in Melbourne, Australia; (c) novel nesting structures, bats roosting under a motorway in Sydney, Australia; (d) highly modified habitats, smooth‐coated otters reestablished along an urban coastline in Singapore; (e) creative solutions, road‐crossing structures to improve connectivity for primates in urban Brazil; and (f) novel landscapes, green roof with an insect hotel.

Small urban spaces can support and sustain populations of native species. Even very small landscape elements, such as solitary trees (Stagoll et al. 2012) or ponds (Calhoun et al. 2014; Hill & Wood 2014), provide critical habitat resources. Many species can inhabit small patches in altered landscapes by adjusting their home range and behaviors or by taking advantage of resources that lie beyond the patch within the urban matrix (Shochat 2004; Wright et al. 2012). In some cases, small urban habitats support comparable populations and species diversity to nonurban areas, are critical to the persistence of local populations, and enhance regional diversity. For example, a comprehensive analysis of 80 ponds in Switzerland not only found little evidence for taxon‐specific species‐area relationships, but the number of species in a set of small ponds was greater than a single large pond of comparable total area (Fig. 1a) (Oertli et al. 2002). Similarly, an assessment of a network of urban grasslands in Australia showed that small grasslands contained unique species not found in larger reserves and thus contributed to the overall biodiversity of the landscape (Kendal et al. 2017). The potential for cumulative biodiversity gains to be made through the management of multiple small urban spaces may also better attract conservation initiatives led by local government or community groups with limited resources. Protecting and enhancing small landscape elements in urban environments through appropriate policy and decision making is therefore critical to maintain native biodiversity in cities and towns.

### Recognizing Unconventional Habitats

Urban areas abound with unconventional habitats: areas originally created for human use that can provide important habitat or resources for native biodiversity. The potential for unconventional habitats is wonderfully diverse, ranging from large spaces such as brownfields, golf courses, and cemeteries (Colding & Folke 2009; Threlfall et al. 2015; Gilchrist et al. 2016; Gallo et al. 2017) to smaller pockets such as roadsides or cavities within buildings and infrastructure (Fig. 1b & 1c) (Ray & George 2009; Maclagan et al. 2018). For example, wetlands constructed to trap sediments and treat stormwater before it enters creeks and rivers are readily inhabited by a variety of native species (Hassall & Anderson 2015). Similarly, public and private gardens often provide novel resources that might not otherwise exist in the urban landscape (Davies et al. 2009; Schlaepfer et al. 2011; Chalker‐Scott 2015). For example, 2 endangered butterfly species (genus *Eumaeus*) persist in urban areas of Mexico and the United States because their favored cycad host plant is a popular ornamental species in urban gardens, parks, and roadsides (Ramírez‐Restrepo et al. 2017), while the number and diversity of urban street trees has contributed to large range extensions of the nationally vulnerable grey‐headed flying fox (*Pteropus poliocephalus*) in several Australian cities (Williams et al. 2006). In Singapore, smooth‐coated otters (*Lutrogale perspicillata*) have reestablished resident populations along the urban coastline after a 30‐year absence (Fig. 1d) (Theng & Sivasothi 2016). Managing native biodiversity in unconventional habitats will depend on developing strong partnerships and collaborations with a range of stakeholders, along with careful evaluation of the species use of, and survival in, these spaces to ensure that they are beneficial in the long term and do not function as ecological traps (Schlaepfer et al. 2002). Ultimately though, managers in urban environments can achieve conservation gains in spaces that might otherwise be ignored by considering how a wider variety of spaces and land uses can benefit biodiversity.

### Developing Creative Actions

There is a growing need to intentionally create conditions for nature to thrive in urban environments (Rosenzweig 2003; Sanderson & Huron 2011). This includes actions to minimize human–wildlife conflict, reduce mortality rates, or provide resources that might otherwise be lacking in urban areas (e.g., feeding or nesting sites). Changing the type of street lighting, for example, can reduce the impact of artificial light on nocturnal species (Lewanzik & Voigt 2017), novel collars can reduce predation of urban wildlife by domestic cats (Calver et al. 2007), and artificial cavities can provide suitable nesting sites for wildlife (Bender et al. 2016; Griffiths et al. 2018). Artificial structures, such as wildlife bridges and tunnels, can also be used to overcome barriers to movement created by urban infrastructure (Fig. 1e). Rope bridges installed as part of the Urban Monkeys Program in southern Brazil provided safe passage for brown howler monkeys (*Alouatta guariba clamitans*), porcupine (*Spiggurus villosus*), and white‐eared opossum (*Didelphis albiventris*) across urban roads (Teixeira et al. 2013). More recently, conservation scientists have advocated for bolder initiatives in urban environments, such as creating habitats on built infrastructure (Fig. 1f) (Williams et al. 2014), recognizing the value of novel ecosystems (Hobbs et al. 2009; Kowarik 2011), and restoring species through reintroduction and translocation (Watson & Watson 2015). If creative actions are to become routine management practice, they must be accompanied by a thorough and coordinated evaluation of their effectiveness. Studies that take an experimental approach to evaluating new methods (e.g., Lewanzik & Voigt 2017; Griffiths et al. 2018; Soanes et al. 2018) will help build an evidence base for urban conservation that can guide managers and practitioners to apply creative practices that promote biodiversity in cities and towns.

### Minimizing Future Impacts

In rethinking urban conservation, one must also have future urban development squarely in sight. Urbanization is accelerating through expansion (Jim 2004; Seto et al. 2011) and densification (Haaland & van den Bosch 2015; Hedblom et al. 2017); the majority of urban growth is predicted to occur in biodiversity hotspots in Asia and Africa (Seto et al. 2012; Schneider et al. 2015; UN DESA 2018). This will place increasing pressure on natural environments, including direct impacts in situ such as habitat loss, fragmentation, and degradation, as well as indirect impacts that can propagate a city's ecological footprint far beyond the immediate area of development (Rees & Wackernagel 1996). Although minimizing the ecological footprint of cities requires sustainability innovations beyond the scope of this paper, we point to existing scientific evidence and tools that can be used to build urban environments with improved outcomes for biodiversity. At the landscape scale, systematic conservation planning can be used to plan new cities or suburbs that maximize development objectives while avoiding areas critical for biodiversity. This approach explicitly quantifies and maps the relative biodiversity value of different areas across the landscape (e.g., based on modeled habitat quality for target species or expert opinion) to help stakeholders visualize, understand, and deliberate the merits of multiple urban development options. Bekessy et al. (2012) showed how systematic planning methods can aid the community and decision makers in making informed and intelligent trade‐offs, such as designating development zones in areas of lower biodiversity value in western Melbourne (Australia), while Ground et al. (2016) illustrated the value of urban and peri‐urban conservation planning for the conservation of grassland ecosystems in the rapidly urbanizing eThekwini Municipal Area of South Africa. Analyses that take approaches that address the entirety of a landscape will be particularly useful for comparing oft‐debated development alternatives, such as sharing versus sparing (Lin & Fuller 2013) or sprawl versus densification (Rebelo et al. 2011), as well as the likely outcomes of sustainable development initiatives (Güneralp et al. 2017). Considering the site‐level scale (i.e., 10s to 1000s of m^2^), evidence‐based urban design principles can help develop neighborhoods that are more sensitive to biodiversity (Milder 2007; Hostetler & Drake 2009; Marshall 2013; Ikin et al. 2015; Garrard et al. 2017). These synthesize the growing body of urban ecological research to show how protecting and increasing habitat, facilitating dispersal and ecological processes, minimizing threats, and promoting positive human–nature interactions can all be achieved in urban developments. Garrard et al. (2017) proposed the framework of “biodiversity sensitive urban design” to guide the implementation of these principles during the design, construction, and postconstruction phases of development. A key advantage of this approach is the emphasis on ensuring the persistence of biodiversity within urban settings, in contrast to offsetting (Chee 2015), which is unlikely to provide fair and adequate compensation for urbanization impacts (Coker et al. 2018). Regardless of scale, achieving better biodiversity outcomes in future urban developments will depend on forward planning and collaborative partnerships among the community, government, ecologists, planners, engineers, and architects to develop co‐created solutions.

## Urban Conservation as an Opportunity Waiting

“The problems of urban conservation are not insurmountable, but success requires a careful start” (Dearborn & Kark 2010). Conserving native biodiversity is both important and achievable in cities and towns. There is mounting evidence that the public support the conservation of urban biodiversity (Chen & Jim 2010; Olive 2014), including the importance of interactions with charismatic species (Savard et al. 2000; Stokes et al. 2010) and the cultural significance of urban nature (Cocks & Dold 2006; MSDI 2015). The presence of biodiversity in cities also benefits people, improving human health and well‐being through connection to nature (Fuller et al. 2007; Shanahan et al. 2015). Community engagement can also boost biodiversity conservation: the urban community was instrumental in documenting the return of smooth‐coated otters to Singapore (Theng & Sivasothi 2016) and the installation and monitoring of rope bridges for arboreal mammals in southern Brazil (Teixeira et al. 2013). However, long‐held perceptions that undervalue urban environments undermine opportunities for conservation. The messages we have highlighted here update the narrative of urban biodiversity conservation, enabling researchers, policy makers, planners, and practitioners to act based on scientific evidence and tools. Although our list is not exhaustive, tackling current misconceptions represents a critical step in moving toward effective conservation action in urban spaces. Recognizing the value of small spaces and unconventional habitats for native species, and the potential for creative conservation opportunities, opens up new avenues for managers in urban environments and will lead to better conservation outcomes. For example, researchers in Germany reintroduced grassland species to urban wasteland lots, taking advantage of these small, unconventional spaces to create novel ecosystems that met conservation goals (Fischer et al. 2013). Further, the experimental approach allowed the researchers to identify the most successful and cost‐effective methods and provide guidance to land managers. Re‐imagining urban spaces and proposed developments as opportunities for conservation gains rather than as derelict, modified habitats helps empower communities and local managers to take positive actions for biodiversity on a local scale. In this way, overcoming the misconceptions that constrain conservation action for biodiversity in urban environments will ultimately benefit both urban biodiversity and the humans that live in cities.
